# Direct Observation of Biaxial Nematic Order in Auxetic Liquid Crystal Elastomers

**DOI:** 10.3390/ma16010393

**Published:** 2022-12-31

**Authors:** Zhenming Wang, Thomas Raistrick, Aidan Street, Matthew Reynolds, Yanjun Liu, Helen F. Gleeson

**Affiliations:** 1 School of Physics and Astronomy, University of Leeds, Leeds LS2 9JT, UK; 2 Department of Electrical and Electronic Engineering, Southern University of Science and Technology, Shenzhen 518055, China

**Keywords:** auxetic material, liquid crystal elastomer, biaxial nematic, biaxial order, negative Poisson ratio, Raman scattering, conoscopy, mechanical Frèedericksz transition

## Abstract

Auxetic materials exhibit a negative Poisson’s ratio, i.e., they become thicker rather than thinner in at least one dimension when strained. Recently, a nematic liquid crystal elastomer (LCE) was shown to be the first synthetic auxetic material at a molecular level. Understanding the mechanism of the auxetic response in LCEs is clearly important, and it has been suggested through detailed Raman scattering studies that it is related to the reduction of uniaxial order and emergence of biaxial order on strain. In this paper, we demonstrate direct observation of the biaxial order in an auxetic LCE under strain. We fabricated ~100 μm thick LCE strips with complementary geometries, exhibiting either planar or homeotropic alignment, in which the auxetic response is seen in the thickness or width of the sample, respectively. Polarized Raman scattering measurements on the planar sample show directly the reduction in the uniaxial order parameters on strain and suggest the emergence of biaxial order to mediate the auxetic response in the sample thickness. The homeotropic sample is studied via conoscopy, allowing direct observation of both the auxetic response in the width of the sample and increasing biaxiality in the LCE as it is strained. We verified that the mechanism of the auxetic response in auxetic LCEs is due to the emergence of the biaxial order and conclude such materials can be added to the small number of biaxial nematic systems that have been observed. Importantly, we also show that the mechanical Frèedericksz transition seen in some LCEs is consistent with a strain-induced transition from an optically positive to an optically negative biaxial system under strain, rather than a director rotation in a uniaxial system.

## 1. Introduction

Auxetics are a remarkable class of materials which display anomalous expansion in at least one dimension along the direction perpendicular to an applied extension, i.e., they have a negative Poisson’s ratio [1]. There is a zoo of associated properties that are enhanced compared to conventional materials, including indentation resistance, shock absorbance and delamination resistance, making auxetic materials exciting contenders in application areas as diverse as biomedical sciences, aerospace, architecture, and sporting equipment [2,3,4,5,6]. The first synthetic auxetic material that responded at a molecular level was a liquid crystal elastomer (LCE), discovered in 2018 [7], some 30 years after the effect was observed in porous (so-called re-entrant) auxetics such as polyurethane foam [8]. In nature, auxetic materials are also known in crystalline materials [9,10,11,12], e.g., the α-cristobalite which is a silicon dioxide polymorph; additionally, biological materials such as cat skin [13] and the Achilles tendon [14] have been found to display an auxetic response. The mechanisms responsible for the auxetic response in re-entrant materials are well known and are based on their geometry; many are designed with porous, honeycomb structures [5,15,16] that ‘unfold’ under strain. The advantages of auxetic LCEs are clear; they respond on a molecular level and so are not limited to bulk applications, they are transparent, they are readily synthesized, and as they are not porous, they do not have the inherent mechanical weakness that can plague re-entrant auxetic structures. However, to take full advantage of auxetic LCEs, it is critical to have a detailed understanding of the underlying physics that results in their negative Poisson’s ratio. This paper shows through both direct and indirect observation that the emergence of biaxial order is unambiguously associated with the auxetic response in nematic LCEs.

Many ideas have been put forward for realizing molecular auxeticity [17,18], and liquid crystalline polymers have long been recognized as promising materials [19,20,21,22,23]. For example, He et al. [21,22] studied a main-chain liquid crystalline polymer by X-ray diffraction and suggested a potential auxetic response from the rotation of mesogenic units into the direction transverse to the initial nematic director under the strain [22]. However, only a positive Poisson’s ratio was recorded for this material. The first experimental report of a negative Poisson’s ratio in a liquid crystal system was in the nematic elastomer synthesized by Mistry et al. [7]. For their side-chain LCE, the auxetic response occurs above a threshold strain along an axis perpendicular to both the initial director and the extension direction. Detailed investigations showed that the LCE director rotated discontinuously via a so-called mechanical Frèedericksz transition (MFT) [24], rather than the continuous rotation associated with the semi-soft elastic (SSE) response seen in most LCEs [25,26]. Further, volume is conserved during strain, i.e., no porosity emerges during the deformation process, suggesting quite a different mechanism behind the auxeticity than in re-entrant auxetics. The significant changes in birefringence of the auxetic LCE seen under strain indicated that there was a dramatic change in order associated with the auxetic response, hinting at an order-moderated mechanism. Raistrick et al. [27] explored the order of the LCE under strain using polarized Raman spectroscopy (PRS), a technique that allows both uniaxial and biaxial order parameters to be inferred. Fitting the Raman depolarization data with a biaxial model strongly suggested that the auxetic response was related to out-of-plane rotations of the mesogenic units and the emergence of biaxial order.

Raistrick et al.’s study stopped short of *proving* that the emergence of biaxiality was implicit in auxetic response of the LCE. Herein, we have fabricated auxetic LCE samples using the same monomers as Mistry [7,24], but the different proportions result in a material with subtly different mechanical properties, one of which is a much lower auxetic strain threshold. The LCE samples are synthesized in planar and homeotropic geometries and the stress-induced mechanical deformation of each sample is characterized to show the auxetic behavior. PRS is employed to determine the uniaxial and biaxial order parameters in the planar sample, while conoscopy on the homeotropic sample directly shows the emergence of the biaxiality in the nematic LCE.

## 2. Materials and Methods

### 2.1. Sample Fabrication

**Figure 1:**
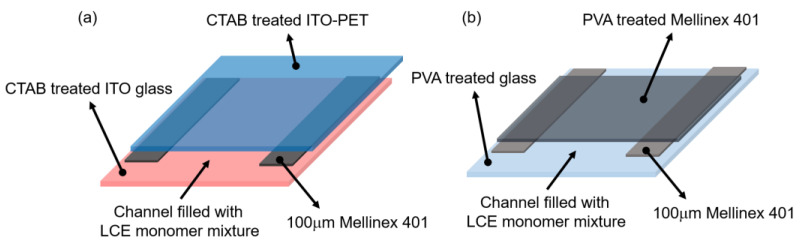
A schematic showing the assembled LCE mold used to fabricate LCEs with (**a**) homeotropic alignment and (**b**) planar alignment.

The LCE samples were made by photopolymerizing the nematic precursor mixture in a mold based on a conventional liquid crystal device, shown schematically in Figure 1. The empty devices were assembled using one glass and one polymer substrate, the latter allowing ease of extraction of the LCE film after polymerization. The planar samples were constructed as described previously [7,24,27], with 100 μm thick Melinex401 film (DuPont Teijin Films) used as spacers. Excellent monodomain alignment is achieved by spin-coating the inner surface of the substrates with a 0.5 wt.% polyvinyl alcohol (PVA) solution which is uniaxially rubbed when dry. The process was modified slightly for fabrication of the homeotropic sample where the application of an electric field was required to supplement the homeotropic surface alignment to achieve a uniform, 100 μm thick, monodomain sample. For those samples, the glass substrate was coated with indium–tin oxide (ITO) (Xinyan Technology Ltd., China) and the polymer substrate was ITO-coated PET film (ITO-PET) (Sigma-Aldrich, Unite States) spin-coated with 0.5 wt.% cetyl trimethyl ammonium bromide (CTAB) (MP Biomedicals, France) solution and left unrubbed. The molds were capillary-filled with the monomer mixture described in the following section.

### 2.2. LCE Synthesis

**Figure 2:**
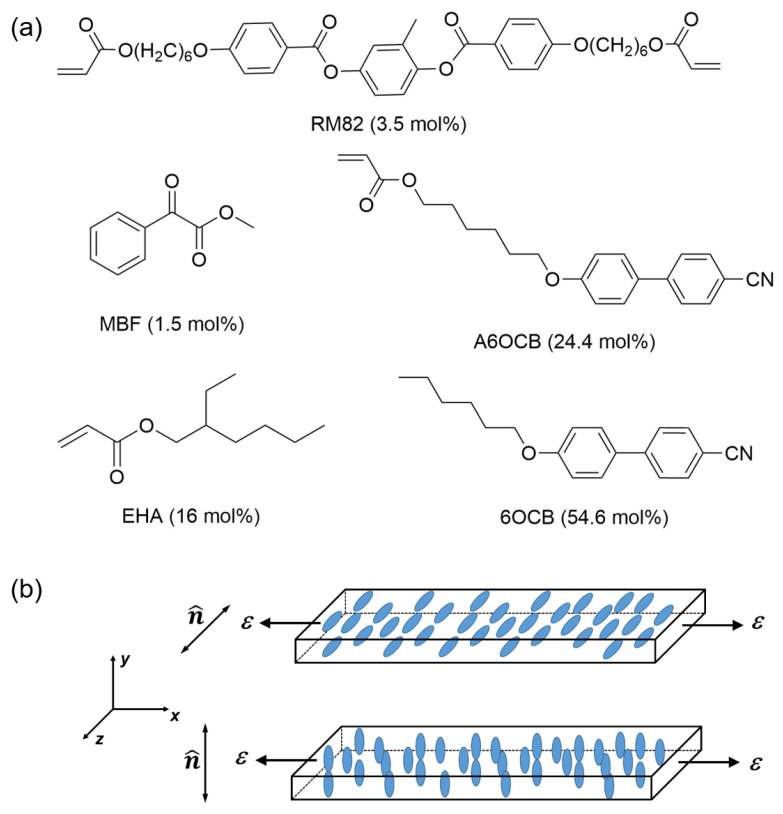
(**a**) The molecular structures of the LCE monomers with proportion in the precursor mixture indicated. The composition is similar to [7]. (**b**) Diagram describing the undeformed sample geometry with the liquid crystal director parallel to the *z*-axis for the planar LCE and to the *y*-axis for the homeotropic LCE. In this work, deformations (*ε*) are applied along the *x*-axis.

The LCE was created using the method described by Mistry et al. [7]. The chemical compounds shown in Figure 2 were mixed in the proportions indicated to form the nematic LCE precursor. The 4′-hexyloxybiphenyl (6OCB) is a nonreactive mesogen included to broaden the nematic phase range of the precursor. The 6-(4-cyano-biphenyl-40-yloxy)hexyl acrylate (A6OCB) is a monofunctional reactive material that forms the LCE mesogenic side groups, while the bifunctional 1,4-bis-[4-(6-acryloyloxyhex-yloxy)benzoyloxy]-2-methylbenzene (RM82) is a mesogenic crosslinker. 2-Ethylhexyl acrylate (EHA) was introduced to increase the flexibility of the polymer backbone, helping to reduce the glass transition temperature to below room temperature and methyl benzoylformate (MBF) was used as the UV-photoinitiator. A6OCB, 6OCB, and RM82 were obtained from Synthon Chemical GmbH (Bitterfeld-Wolfen, Germany), while EHA and MBF were from Sigma Aldrich (Gillingham, UK).

The mesogenic compounds were mixed by heating to 120 °C while stirring at 200 rpm on a magnetic hot plate for 5 min. The temperature was then decreased to 35 °C and the EHA and MBF added, stirring for a further 2 min. The LCE monomer mixture was filled into the molds via capillary action in its isotropic phase (at 35 °C) and left for around 20 min to cool to room temperature. The mesogens align with the alignment layers as the temperature decreases into the nematic phase. For the homeotropic sample, conductive tapes attached onto the edges of the mold allowed an alignment voltage of 40 V_rms_ at 1 kHz to be applied, enhancing the surface alignment mechanism. Once aligned, the molds were placed under a low intensity UV florescence light source (2.5 mW/cm^2^) for 2 h to cure, with the field maintained in the case of the homeotropic sample.

After curing, the polymer substrate was carefully peeled away. Placing the exposed sample on the glass substrate into methanol caused the sample to swell slightly and delaminate at the edges. Flat-tipped tweezers were used to peel off the sample completely. Once separated, the unreacted 6OCB in LCE was washed away by leaving it in dichloromethane (DCM) solution (30% in methanol) overnight. After washing, the LCE film was hung in a beaker at 60 ℃ for 2 h to dry. Table 1 shows the proportions of the components for the monomer mixture and those in the final LCE sample.

### 2.3. Mechanical Deformation Measurements

Mechanical measurements were undertaken using bespoke equipment that was built in-house, comprising two actuators and a load cell enclosed in a temperature-controlled environment. The apparatus is equipped with optics that allows polarizing microscopy of the LCE under strain. The full specification of the equipment and analysis methodologies, which allow simultaneous optical and mechanical studies of LCEs, are described in previous reports [7,27]. For this work, the original gap between the two actuators was 10 mm and the LCE samples were loaded at room temperature. The samples were stretched using strain steps of 0.5 mm and a waiting time 10 min for each step. The geometries of the LCE films and direction of the strain are shown in Figure 2b.

### 2.4. Order Parameter Measurements

Polarized Raman spectroscopy (PRS) can be used to determine a number of the order parameters of liquid crystalline and polymeric systems by analyzing the intensity of light scattered via the Raman mode [27]. The Raman scattering process is the inelastic scattering of light, related to rotational and vibrational molecular motions. The Raman scattered light has a frequency shift in comparison to the incident light and the extent of the shift is related to a specific molecular vibration or rotation mode. The intensity of a specific Raman mode is proportional to the square of the differential polarizability tensor [28]; the tensorial nature allows the anisotropic properties and order parameters of a liquid crystal to be captured. PRS is a particularly useful technique in this regard as it allows the 2nd and 4th rank-order parameters to be determined. The procedure to determine uniaxial and biaxial order parameters in LCEs has been described previously [26]; however, the key equations and experimental technique are both briefly described herein.

Order parameters are determined using a monodomain planar sample with a uniform director placed in the *x*–*z* plane. The intensity of a specific vibrational Raman mode is recorded both parallel $I_{∥}$ and perpendicular $I_{⊥}$ to the incident laser polarization. For a uniaxial system with no tilt angle relative to the molecular long axis and no bend angle present within the mesogen, $I_{∥}$ and $I_{⊥}$ depends primarily on the 2nd and 4th rank uniaxial order parameters as shown in equations (1) and (2) [28]:

(1) $$\begin{array}{c} \;\;\;\;I_{∥}=\frac{2}{15}\left(5\left(1+2r+3r^{2}\right)+{\left(r-1\right)}^{2}\right)-\frac{1}{42}\langle P_{200}\rangle \left(r-1\right)\left(5+9r+6\left(3+4r\right)-\left(r-1\right)\right)\left(1+3\cos2\theta \right)\\ +\frac{1}{70}\langle P_{400}\rangle {\left(r-1\right)}^{2}\left(9+20\cos2\theta +35\cos4\theta \right) \end{array}$$

(2) $$I_{⊥}=\frac{4}{15}{\left(r-1\right)}^{2}+\frac{4}{21}\langle P_{200}\rangle {\left(r-1\right)}^{2}-\frac{1}{70}\langle P_{400}\rangle {\left(r-1\right)}^{2}\left(-3+35\cos4\theta \right)$$

where r is the differential polarizability ratio relating to the differential polarizability tensor, θ is the laboratory angle of the sample, $\left\langle P_{200}\right\rangle$ and $\left\langle P_{400}\right\rangle$ are, respectively, the 2nd and 4th rank uniaxial order parameters which are given by [27]:

(3) $$\langle P_{200}\rangle =\frac{1}{2}\left\langle (3cos^{2}\left(\beta \right)-1)\right\rangle$$

(4) $$\langle P_{400}\rangle =\frac{1}{8}\left\langle (3-30cos^{2}\left(\beta \right)+35cos^{2}\left(\beta \right))\right\rangle$$

In Equations (3) and (4), β is the Euler angle of the molecular long axis with respect to a chosen frame of reference. The behavior of the biaxial order parameters can be inferred by assuming that the uniaxial order parameters follow Maier–Saupe theory as described in Ref. [26]. For a biaxial system, the intensity of the vibrational Raman mode is dependent on the biaxial order parameters, $\left\langle P_{220}\right\rangle$, $\left\langle P_{420}\right\rangle$, $\left\langle P_{440}\right\rangle$, in addition to the uniaxial order parameters [26]:

(5) $$\begin{array}{l} I_{∥}=\frac{2}{3}\left(1+2r+3r^{2}+{\left(r-1\right)}^{2}\right)-\frac{4}{21}\langle P_{200}\rangle \left(r-1\right)\left(3+4r\right)\left(1+3\cos2\theta \right)\\ \;\;\;\;\;\;+\frac{1}{70}\langle P_{400}\rangle {\left(r-1\right)}^{2}\left(9+20\cos2\theta +35\cos4\theta \right)-\frac{16}{7}\langle P_{220}\rangle \left(r-1\right)\left(3+4r\right)sin^{2}\theta \\ \;\;\;\;\;\;+\frac{24}{7}\langle P_{420}\rangle {\left(r-1\right)}^{2}\left(5+7\cos2\theta \right)sin^{2}\theta +8\langle P_{440}\rangle {\left(r-1\right)}^{2}sin^{4}\theta \end{array}$$

(6) $$\begin{array}{l} I_{⊥}=\frac{4}{15}{\left(r-1\right)}^{2}+\frac{4}{21}\langle P_{200}\rangle {\left(r-1\right)}^{2}-\frac{1}{70}\langle P_{400}\rangle {\left(r-1\right)}^{2}\left(-3+35\cos4\theta \right)+\frac{8}{7}\langle P_{220}\rangle {\left(r-1\right)}^{2}\\ \;\;\;\;\;\;+\frac{6}{7}\langle P_{420}\rangle {\left(r-1\right)}^{2}\left(1+7\cos4\theta \right)+2\langle P_{440}\rangle {\left(r-1\right)}^{2}\sin^{2}2\theta \end{array}$$

where the biaxial order parameters are functions of the Euler angles, α and β [26]:

(7) $$\langle P_{220}\rangle =\frac{1}{24}\left\langle (7\cos^{2}\left(\beta \right)-1\right)\left(1-\cos^{2}\left(\beta \right)\right)\cos\left(2\alpha )\right\rangle$$

(8) $$\langle P_{420}\rangle =\frac{1}{16}{\left\langle (1-\cos^{2}\left(\beta \right)\right)}^{2}\cos\left(4\alpha )\right\rangle$$

(9) $$\langle P_{440}\rangle =\frac{1}{4}{\left\langle (1-\cos^{2}\left(\beta \right)\right)}^{2}\cos\left(2\alpha )\right\rangle$$

PRS was performed using a Renishaw inVia Raman microscope in backscattering geometry with a 532 nm solid state laser at a relative power of 5%, an exposure time of 1 s and 5 accumulations. The settings were chosen in order to minimize fluorescence and avoid damage to the sample while maintaining easily identifiable characteristic peaks. The depolarization ratio, R =$\;\;I_{∥}$/$I_{⊥}$ was measured at increasing sample strains between the unstrained length, *ε_x_* = 0 and *ε_x_* = 1.23 ± 0.05 using Raman measurements made for each strain step at 10° intervals between θ = 0° and θ = 180°.

Herein, order parameters were determined using the 1606 cm^−1^ vibrational mode associated with the C-C stretch of the biphenyl rings of the mesogenic units [28,29]. This vibrational mode was selected over the 2250 cm^−1^ vibrational mode, which is associated with the C≡N stretch, as it more closely fits the assumptions required to determine order parameters via Raman spectroscopy [30], namely, that the selected vibrational mode is (*i*) cylindrically symmetric and (*ii*) parallel to the long axis of the mesogenic unit [30].

### 2.5. Conoscopy Measurement

Conoscopy was performed using a Leica DM 2700P polarizing microscope in transmission mode under cross-polarized conditions. The microscope was equipped with a 0.9 numerical aperture (NA) condensing lens and a 0.95 NA 80× Leitz microscope objective. To study the conoscopic patterns, a Bertrand lens was inserted between the microscope objective and the eyepiece. The conoscopic patterns were captured with a Nikon D3500 camera. The homeotropic sample is held under strain with Kapton tape and allowed to stress relax for 2 minutes before the image of the conoscopic pattern is recorded. The strain is applied parallel to the analyzer of the cross-polarized microscope and measured with digital calipers. The optical sign of the conoscopic figures was determined by inserting a λ wave-plate and observing the coloration of the grey regions near the melatopes of the acute bisetrix conoscopic figure obtained when the system is rotated to the 45° position. The change in color of the first order ‘grey’ regions of the conoscopic figure indicates whether the system is optically positive or negative [31,32]. A positive uniaxial system produces blue coloration along the direction of the λ wave plate and yellow coloration perpendicular to it [31]. The opposite is true for a negative uniaxial system.

## 3. Results and Discussion

### 3.1. Physical Properties and Mechanical Measurements

**Figure 3:**
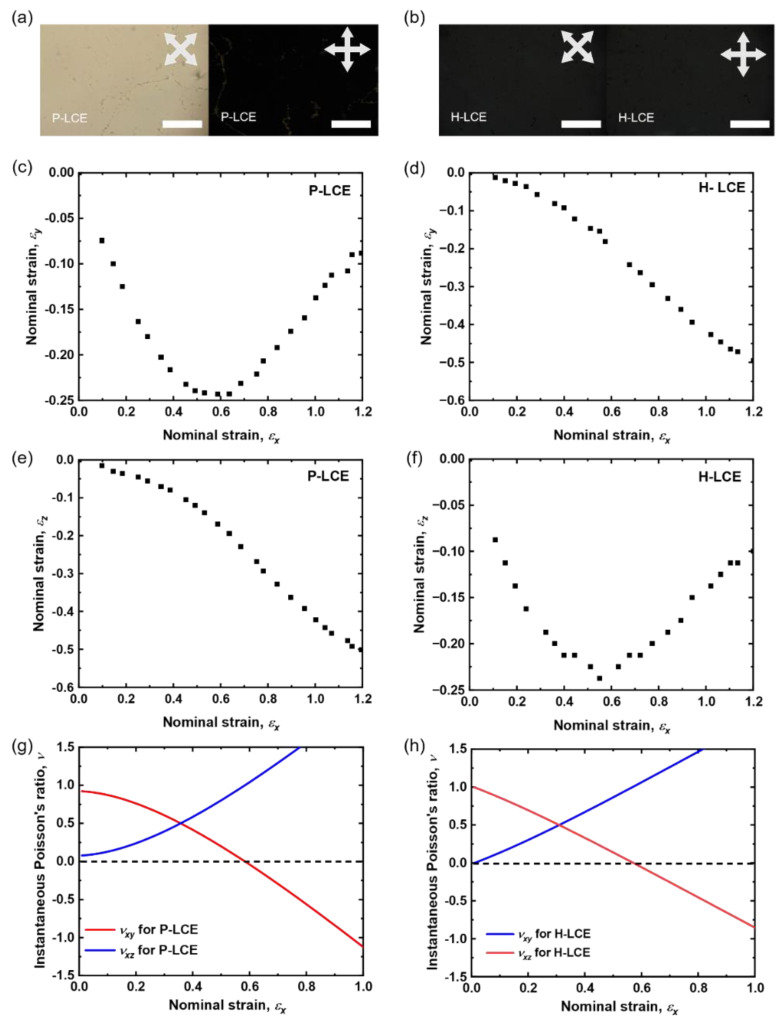
The POM images for (**a**) P-LCE and (**b**) H-LCE. The scale bar is 20 μm and the white arrows indicate the crossed polarizers. The *ε_x_*–*ε_y_* strains measured for (**c**) P-LCE and (**d**) H-LCE, the *ε_x_*–*ε_z_* strains measured for (**e**) P-LCE and (**f**) H-LCE and instantaneous Poisson’s ratio, ν *_xy_* and ν *_xz_* measured for (**g**) P-LCE and (**h**) H-LCE.

The quality of the alignment of the mesogens in the LCE films was monitored using polarizing optical microscopy (POM). The POM images of LCE films with planar (P-LCE) and homeotropic alignment (H-LCE) are shown in Figure 3a. The figure shows that for the P-LCE, when the director orientation is parallel to the crossed polarizers there is an excellent dark state, while at 45° there is a uniform bright state. The uniform high contrast between the two images of the P-LCE illustrates the high quality of the monodomain alignment and the anisotropy of the P-LCE sample. For the H-LCE, there is no light transmitted for either orientation, indicating excellent homeotropic alignment.

For the mechanical measurements, the strains *ε_x_* and *ε_z_* were determined by analysis of photographs of the samples, while *ε_y_* was inferred using the conserved volume conditions known for these samples [7,26]. Figure 3c,e show the behavior of *ε_y_* and *ε_z_* for a strain in the *x*-direction (*ε_x_*), measured for the P-LCE sample. The deformations of the P-LCE sample in the *x–y* and *x–z* planes are highly anisotropic. For the deformations in the *x–z* plane, *ε_z_* decreases with increasing *ε_x_*, with a softening of the sample as was seen for the related LCE in previous work [26]. The deformation of the P-LCE in the *x–y* plane is highly anomalous; *ε_y_* approaches a minimum when *ε_x_* is ~0.58 ± 0.05 and then increases to almost the original sample thickness. The auxetic behavior of the P-LCE is clear; the sample gets thicker in the *y*-direction, perpendicular to *ε_x_*_,_ above strains of ~0.58 ± 0.05. The Poisson’s ratio, ν, can be calculated for each deformation direction as follows. All the strains shown in Figure 3 are engineering strains. By converting them into true strains through *ε*_true_ = ln (*ε*_engineering_ + 1) and fitting a polynomial, the Poisson’s ratio, ν = − (d*ε_trans_*/d*ε_expan_*), i.e., the ratio of the relative deformation in the transverse direction of expansion to the relative expansion [1], can be calculated. Here, the deformations in *y*- and *z*-directions are the transverse deformations and the strain in the *x*-direction is the relative expansion. The Poisson’s ratio of the P-LCE is shown in Figure 3g; it is always positive for deformations in the *x–z* plane, while in the *x–y* plane there is a threshold strain, *ε_x_* ~0.58 ± 0.05 beyond which the Poisson’s ratio becomes negative and the system is therefore auxetic.

Figure 3d,f show the corresponding behavior of the *ε_y_* and *ε_z_* strains for the H-LCE sample. Now, the auxetic response occurs in *x*–*z* plane, *ε_z_* reaching a minimum when *ε_x_* is ~0.56 ± 0.05 and then increasing. The threshold strain for the auxetic behavior of the H-LCE is ~0.56 ± 0.05 (seen from Figure 3h as the point where the Poisson’s ratio becomes negative), in excellent agreement with that of the P-LCE. In each case, the direction of the auxetic response is perpendicular to the direction of both the strain and the initial director alignment direction.

**Figure 4:**
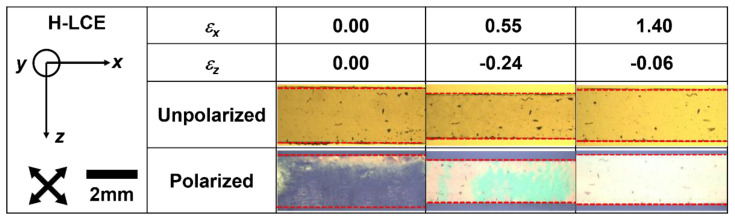
Photomicrographs of the H-LCE during mechanical deformation in the *x–z* plane. The red dashed lines clarify the positions of the sample edges. When *ε_x_* is ~0.55, the width of the LCE is a minimum, beyond which it clearly increases with further strain, i.e., it shows an auxetic response. The H-LCE sample has almost regained its original width at a strain of ~1.4.

One key advantage of the homeotropic geometry is that it allows direct and straightforward observation of the auxetic response of the LCE. Figure 4 shows photomicrographs of the H-LCE sample at various strain steps (see Supplementary Materials for a video of the full bright-field experiment). The sequence shows the width change of the sample in z-direction as the sample extends in the x-direction. When the strain reaches ~0.55, the width of the sample is a minimum and beyond this it increases: a direct demonstration of the auxetic behavior of the H-LCE sample.

### 3.2. Order Parameter Measurements

**Figure 5:**
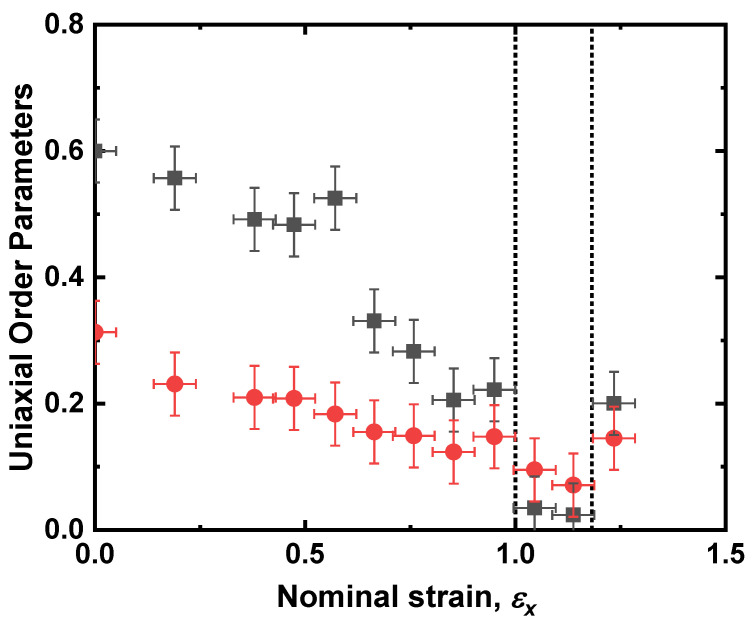
The uniaxial order parameters $\left\langle P_{200}\right\rangle$ (black squares) and $\left\langle P_{400}\right\rangle$ (red circles) as a function of the strain of the sample. The strain domain denoted by the vertical dashed lines shows the region in which $\left\langle P_{200}\right\rangle$ appears to be lower than $\left\langle P_{400}\right\rangle$.

Raman measurements of the uniaxial order parameters, $\left\langle P_{200}\right\rangle$ and $\left\langle P_{400}\right\rangle$, taken for the planar sample are shown in Figure 5. The initial (unstrained) values are $\langle P_{200}\rangle =0.60\pm 0.05$ and $\langle P_{400}\rangle =0.31\pm 0.05$, very similar to those determined for a related LCE [26]. $\left\langle P_{200}\right\rangle$ reduces rapidly from its initial value to effectively zero at a strain of *ε_x_* = 1.0 and $\left\langle P_{400}\right\rangle$ reduces more slowly to a minimum of $\langle P_{400}\rangle =0.07\pm 0.05$ at a strain of *ε_x_* = 1.14 ± 0.05. Note that in the region where $\left\langle P_{200}\right\rangle$ is effectively zero, indicated by the vertical dashed lines in Figure 5, the value of $\left\langle P_{400}\right\rangle$ deduced using the uniaxial model is greater than that of $\left\langle P_{200}\right\rangle$.

**Figure 6:**
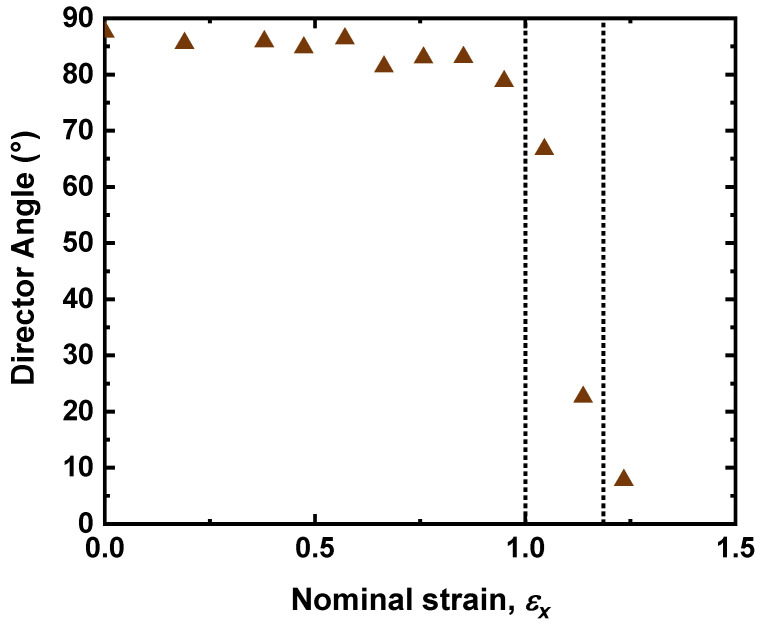
The director angle with respect to the axis of deformation (long axis of the sample) as a function of the strain of the P-LCE sample. The dotted region is the same as Figure 5; in this region the director rapidly reorients to align with the axis of deformation.

The PRS method also allows for measurement of the director angle in the plane of the sample, initially set to be at 88 ± 1° to the long axis of deformation by the rubbing direction of the planar alignment. Figure 6 shows that the director remains constant with strain until *ε_x_* ~1.0, the regime where $\langle P_{200}\rangle \sim 0$. At this strain the director rapidly reorients to align with the axis of deformation, a phenomenon known as a mechanical Fréedericksz transition (MFT) [23,26]. For this LCE, the MFT occurs at a threshold strain of *ε_x_* ∼ 1.0, much later in the strain regime than the auxetic threshold, in contrast to the related LCE reported previously [7,26] where the MFT was approximately coincident with it. This point is returned to later.

**Figure 7:**
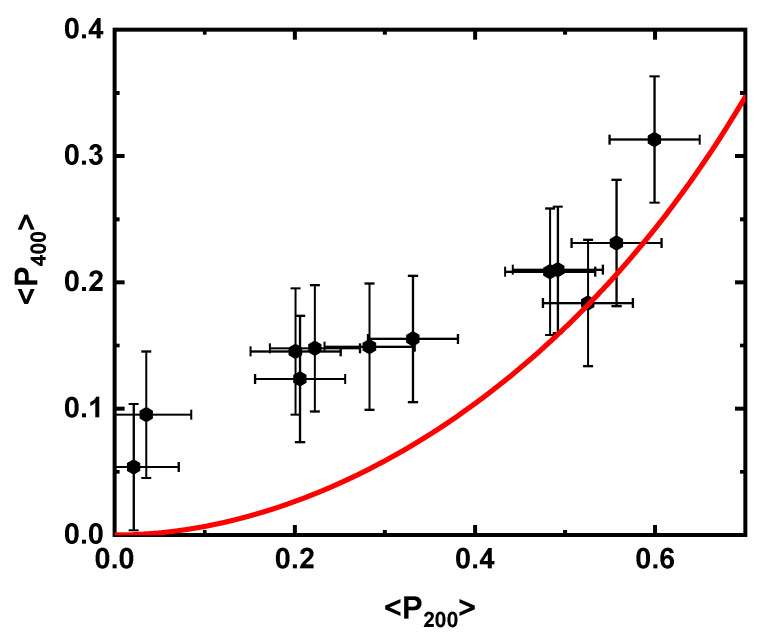
The values of $\left\langle P_{400}\right\rangle$ plotted as a function of $\left\langle P_{200}\right\rangle$ compared to the theoretical prediction of Maier–Saupe theory (red line). A clear deviation can be seen at higher strain (lower $\left\langle P_{200}\right\rangle$ and $\left\langle P_{400}\right\rangle$ values).

**Figure 8:**
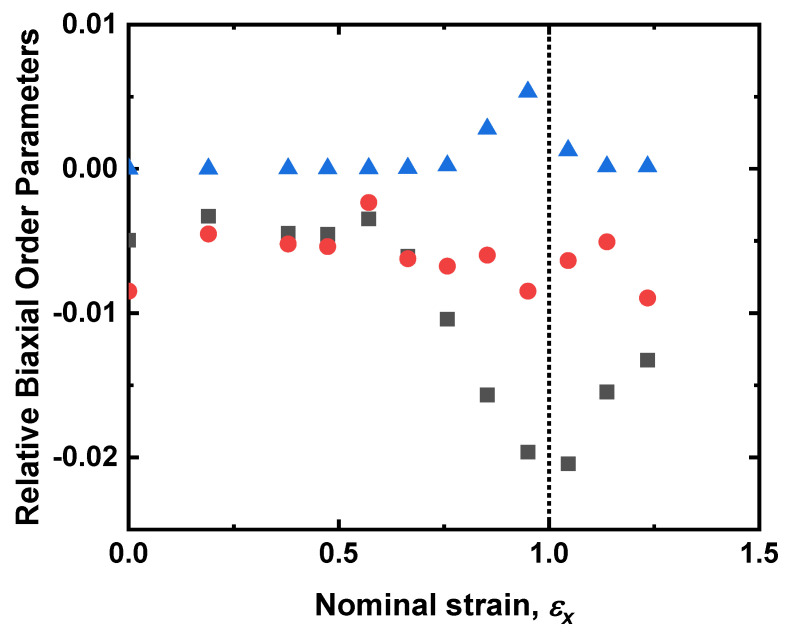
The biaxial order parameters ⟨*P*_220_⟩ (grey squares), ⟨*P*_420_⟩ (red circles) and ⟨*P*_440_⟩ (blue triangles) as a function of strain. The dotted line denotes where the maximum value of ⟨*P*_440_⟩ is seen, a point that coincides with the MFT of this material.

The apparent reduction of $\left\langle P_{200}\right\rangle$ to values below $\left\langle P_{400}\right\rangle$ is an interesting result as it appears to violate Maier–Saupe theory, a mean-field treatment of the long-range intermolecular potential which has had enormous success in describing liquid crystal systems [33]. The deviation from Maier–Saupe theory can be seen in Figure 7. Such a deviation can be assumed to be attributed to the emergence of biaxial order and values for the biaxial order parameters can be generated as follows [26]. The value of $\left\langle P_{200}\right\rangle$ is used to generate idealized values of $\left\langle P_{400}\right\rangle$ in accordance with Maier–Saupe theory, then the biaxial order parameters are allowed in the fit to the PRS data. Figure 8 shows the deduced behavior of $\left\langle P_{220}\right\rangle$, $\left\langle P_{420}\right\rangle$, and $\left\langle P_{440}\right\rangle$, over the whole extension regime. The fourth-order terms, $\left\langle P_{420}\right\rangle$ and $\left\langle P_{440}\right\rangle$ change little; the former showing no particular trend and the latter remaining close to zero up to a strain of *ε_x_* = 0.85 ± 0.05, where a positive value is observed with a maximum value at *ε_x_* = 0.95 ± 0.05. $\left\langle P_{220}\right\rangle$ shows the biggest variation, with significant changes for strains of beginning at ε=0.6, where the value of $\left\langle P_{220}\right\rangle$ rapidly becomes maximally negative, $\left\langle P_{220}\right\rangle$ = 0.02 ± 0.005 at a strain of *ε_x_* = 1.05 ± 0.05. The increasingly negative value seen in $\left\langle P_{220}\right\rangle$ is indicative of an increasing population of molecules aligned with the strain axis [26]. In contrast, the fourth-order biaxial order parameters are expected to drive a distribution of molecules aligned out of plane in the direction that the auxetic response is observed [26].

It is clear that the emergence of biaxial order is inferred by the PRS analysis both for this system and for that previously studied [26], where calculation of the orientational distribution function showed a significant population of out of plane mesogens, suggesting a mechanism for the auxetic response. However, the values determined for the biaxial order parameters using this approach are relative [26] rather than specific. This means that it is possible that the magnitudes of the values generated may be unrealistic; additional proof is required that the size of the biaxial response is sufficiently large to explain the strong auxetic response seen in these materials. This point is considered in the next section of the paper.

### 3.3. Conoscopy

**Figure 9:**
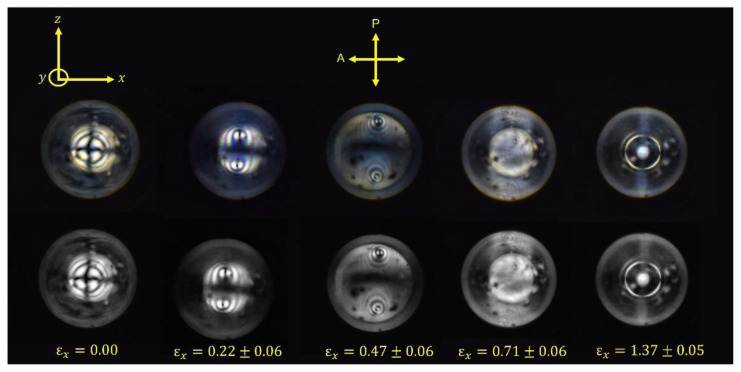
Conoscopic patterns for the homeotropically aligned LCE. The initial nematic director is aligned along the y-axis and strains are applied along the x-axis with a polarizer and analyzer aligned along the x- and z-axis respectively. The development of two melatopes with applied strain (increasing strain from the left to the right of the figure) is indicative of biaxiality within the system. The bottom row shows grey-scaled images of the top row.

The PRS data for the P-LCE clearly suggests that biaxiality is induced in the system through the application of a strain perpendicular to the initial nematic director. Conoscopy can be used to provide a direct insight into the quality of the uniaxial alignment of the (initially) homeotropic LCE as well as allowing a direct observation of biaxiality [30] if it is induced in the system via the application of strain. Conoscopy was performed as described in Section 2.5 on the H-LCE sample, Figure 9.

Figure 9 shows clearly that the unstrained, homeotropically aligned LCE has uniaxial symmetry with zero pre-tilt as evidence by a dark symmetric “Maltese cross” pattern in the center of the conoscopic image [33]. On the application of a strain of *ε_x_* = 0.22 ± 0.06 along the *x*-axis, the LCE sample has clearly become biaxial as evidenced by the two melatopes in the image [34] which suggest that the sample is being investigated perpendicular to the acute bisectrix [35]. On increasing strain, the melatopes separate further apart, and higher order isochromes become visible, both of which are indicative of an increasing difference in the retardation of polarized light traveling through the sample. From Figure 9, it can be seen that, in the geometry required for conoscopic investigation of the homeotropic LCE, the distance the light travels through the sample always decreases with applied strain (i.e., light is travelling along *y* which shows non-auxetic behavior). Therefore, the greater separation of the melatopes and the increase in the order of isochromes visible in the conoscopic figures are both indicative of an increase in biaxiality within this system upon increasing applied strain.

**Figure 10:**
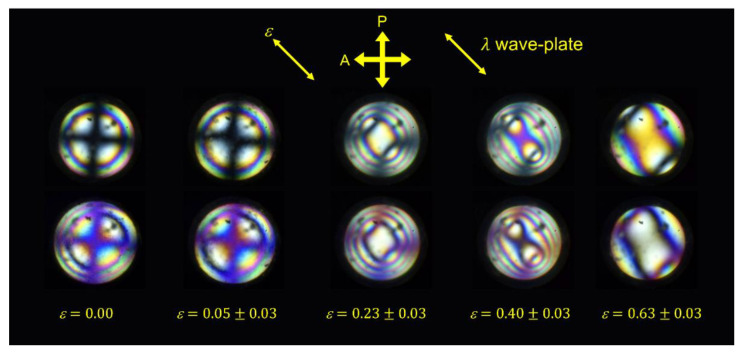
Conoscopic figures for the homeotropically aligned LCE strained at 45° to the crossed-polarizers. The top and bottom rows show the conoscopic images without and with a λ-wave-plate inserted, respectively.

To confirm the optical sign of the biaxial ordering in the LCE, conoscopic observations were performed on a H-LCE sample strained at 45° to the crossed-polarizers. The sample is strained in the *x*-direction and the optical sign is determined through the insertion of a λ wave-plate parallel to the melatopes (NW-SE position), see Figure 10. Figure 10 reveals that the unstrained H-LCE is a positive uniaxial system due to the existence of blue coloration along the direction of the λ wave plate (NW-SE) and yellow coloration perpendicular to it. Even at the very low strain value of *ε* = 0.05 ± 0.03, there is some separation of the isogyres indicating that biaxiality is induced with a very low or no threshold strain. The insertion of a λ wave-plate results in no yellow coloration between the isogyres (possibly due to the small separation between them); however, blue coloration is observed on the outside of the isogyres thus confirming a (small) positive biaxial orientation of the H-LCE. For strains greater than *ε* = 0.23 ± 0.03, the yellow coloration between the two isogyres of the conoscopic figures and blue coloration on the outside edge of the isogyres confirms that the system is biaxial with a positive optical sign.

The information obtained from conoscopy can be considered together with the mechanical behavior of the LCE and the order parameters determined via Raman spectroscopy to give a new insight into the MFT. Up to strains of $\varepsilon_{x}$ = 0.71 ± 0.06, melatopes are visible in the conoscopic figures; however, at $\varepsilon_{x}$ = 1.37 ± 0.05 the melatopes are no longer visible. It is interesting to note that $\varepsilon_{x}$ = 1.37 ± 0.05 is beyond the MFT, i.e., the point where the nematic director apparently rotates. The lack of melatopes in the conoscopic figure would be consistent with a rotation of the nematic director and subsequent collapse of biaxial order. However, the PRS data show that the biaxial order does not collapse discontinuously, so a more likely explanation is that the melatopes are beyond the maximum aperture of the conoscopic experiment and the system remains biaxial. This view is supported by the conoscopic images in Figure 10, which describe a system with growing refractive index, $n_{x}$, in the direction of strain, indicative of *increasing* biaxiality within the system. Consequently, the current model of the MFT as a sudden director rotation to a planar uniaxial conformation [26,36,37] can be seen to be inaccurate and the features of the MFT can instead be explained by the continuously increasing biaxiality along the strain direction. Assuming that the refractive index in the strain direction continues to increase with increasing strain up to the point at which $n_{x}>n_{y}>n_{z}$, there would be a change from an optically positive to an optically negative biaxial system. The ‘director reorientation’ associated with the MFT should therefore be thought of as an apparent rotation; it is better described as the point at which the biaxial indicatrix of the refractive index switches from optically positive to optically negative, i.e., where the minor and major axis of the biaxial nematic directors switch.

This interpretation of the MFT changes our understanding and is entirely consistent with previous findings [24] where a ‘black state’ was observed at the MFT strain in a planar aligned LCE; the transition from an optically positive to negative biaxial system is necessarily through zero (the black state). Further, evidence for this new explanation of the MFT is that if it was in the case that biaxial order collapsed at the MFT, one would expect a return to non-auxetic behavior. This is because biaxiality is implicit in the auxetic response, with a population of molecules in the direction of the auxetic response [27]. Thus, we suggest that the MFT is not at all analogous to a field-induced Frèedericksz transition but is instead an optical effect that results from the growth of biaxiality along the strain direction with the apparent director reorientation occurring when the optical sign of the biaxial indicatrix changes from positive to negative. Such a suggestion is consistent with all previous findings via cross-polarized microscopy, Raman spectroscopy, and the current model for the auxetic response, namely, out-of-plane rotation of the mesogenic units [27].

It is worth making one further observation regarding the ‘MFT’ and the auxetic response of LCEs. In our previous study [26] on a chemically similar LCE, the MFT occurred at ε ∼ 1.16 and the auxetic threshold was at ε ∼ 1.00, i.e., the two thresholds were approximately coincident. In the LCE described in this paper, the MFT is found to occur at ε ∼ 1.0, while the auxetic threshold is at ε ∼ 0.55−0.60. Therefore, it appears that while the optical signature of the MFT is the result of a biaxial response and it is therefore implicit to auxetic behavior as we previously suggested [27], the strain at which the MFT occurs need not be directly related to the auxetic threshold.

## 4. Conclusions

Our motivation for this paper was threefold. Firstly, we aimed to successfully fabricate a high-quality homeotropically aligned auxetic LCE sample with the help of an external electric field and to demonstrate that the auxetic response would occur in the width rather than thickness of the sample for this geometry. Secondly, we aimed to show analogous behavior of this auxetic LCE to that described in previous reports [26], specifically the emergence of biaxial order as an inherent feature of the auxetic response. Last but not least, we wanted to experimentally prove the emergence of significant biaxiality for the auxetic LCE during deformation via conoscopic observation of the homeotropic sample.

The POM figures of the sample, with their high contrast, show excellent, monodomain planar and homeotropic alignment in the unstrained LCE samples. Both types of LCE sample show an auxetic response with threshold strains in excellent agreement, with 0.56 ± 0.05 for the H-LCE and 0.58 ± 0.05 for the P-LCE. The two LCE samples have very different aspect ratios (in the reference frame of the nematic director); however, the onset and extent of the auxetic behavior in the H-LCE and P-LCE is essentially the same. Therefore, we can conclude that (*i*) the auxetic response is not related to the sample aspect ratio and (*ii*) that the auxetic response is related to the geometry of the applied strain with respect to initial nematic director. The auxetic response of the H-LCE can be clearly and directly visualized as a change in the width of the sample.

By measuring the order parameters for the P-LCE via PRS during deformation, the deviation from Maier–Saupe theory was observed for $\left\langle P_{200}\right\rangle$ and $\left\langle P_{400}\right\rangle$ and the biaxial order parameters $\left\langle P_{220}\right\rangle$, $\left\langle P_{420}\right\rangle$, and $\left\langle P_{440}\right\rangle$, were deduced, suggesting the emergence of biaxiality in the initially uniaxial system. The conoscopic images of the H-LCE directly confirm the emergence and increase of biaxiality along the strain axis during the deformation, consistent with the increase in magnitude of $\left\langle P_{220}\right\rangle$ seen from PRS. We have therefore confirmed unequivocally that the emergence of biaxiality is an intrinsic feature of the auxetic response in nematic LCEs. The conoscopic figures reveal that biaxiality is induced even at very small strain values (ε=0.05±0.03) and that for the LCE studied in this paper, it is optically positive up to a strain of ε=0.63±0.03. Considering all of the data together allow us to suggest that the MFT is mis-named and can be understood as a continuous growth of biaxial order along the strain direction rather than a discontinuous rotation of a nematic director. The ‘black state’, a state of zero-retardance seen in auxetic planar LCEs deforming via the MFT can now be understood as the strain at which the refractive index of the biaxial component matches the refractive index of initial alignment (i.e., $n_{x}=n_{y}$ in the reference frame selected herein). This interpretation of the MFT is both intuitive (as a sudden collapse of biaxial order is unphysical) and consistent with the requirement of biaxiality for an auxetic response due to out-of-plane rotations of mesogenic units [27]. Finally, we can conclude that auxetic LCEs under strain are one of very few examples of biaxial nematic liquid crystals.

## Figures and Tables

**Table 1:** Chemical composition of the LCE.

Component	% by mol. of Each Component in the LCE	
	Precursor Mixture	Final LCE
6OCB	54.6	
A6OCB	24.4	55.6
RM82	3.5	8.0
EHA	16.0	36.4
MBF	1.5	

## Data Availability

The data associated with this paper can be found at DOI: https://doi.org/10.5518/1263.

## Supplementary Materials

The following supporting information can be downloaded at: https://www.mdpi.com/article/10.3390/ma16010393/s1, Video S1: Homeotropic sample deformation.
